# Effectiveness and Theoretical Foundations of mHealth Apps for Physical Activity, Healthy Eating, and Weight Loss: Protocol for a Systematic Review and Meta-Analysis

**DOI:** 10.2196/72664

**Published:** 2026-02-17

**Authors:** Victoria Riccalton, Madison Milne-Ives, Rosiered Brownson-Smith, Ananya Ananthakrishnan, Cen Cong, Emily Oliver, Edward Meinert

**Affiliations:** 1 Translational and Clinical Research Institute Newcastle University Newcastle upon Tyne United Kingdom; 2 Centre for Health Technology University of Plymouth Plymouth United Kingdom; 3 Population Health Sciences Institute Newcastle University Newcastle upon Tyne United Kingdom; 4 School of Public Health Department of Primary Care and Public Health Imperial College London London United Kingdom; 5 Gnosis Health Limited Newcastle upon Tyne, England United Kingdom

**Keywords:** digital health, mHealth, behavioral change, review, intention

## Abstract

**Background:**

Obesity is a significant global public health concern. Primary prevention and health promotion to encourage positive health behavior to address obesity could be delivered via mobile health (mHealth), but evidence of apps improving health outcomes over sufficient time frames to be clinically meaningful is limited. mHealth interventions for physical activity, healthy eating, and weight loss typically prioritize intention as the primary driver of behavior. This may limit their impact, as intention does not consistently translate into behavior.

**Objective:**

This review updates a previous systematic review on the effectiveness of mobile apps for health behavior change while narrowing the scope to weight management interventions to enable a more focused analysis. Our primary objective is to investigate the effectiveness of mHealth apps in improving health behaviors with respect to physical activity and healthy eating and to explore the inclusion of behavioral theories and behavior change techniques and the evidence for their effectiveness.

**Methods:**

This protocol follows the PRISMA-P (Preferred Reporting Items for Systematic Reviews and Meta-Analysis Protocols) checklist, and the review will be structured using the PRISMA 2020 statement. Nine databases (PubMed, EMBASE, CINAHL, APA PsycINFO, Cochrane Library, SPORTDiscus, SCOPUS, Web of Science, and Science Direct) will be searched for studies reporting evaluation of the impact of mHealth interventions on weight loss, healthy eating, or physical activity outcomes. EndNote 21 software will be used for deduplication and initial screening, followed by manual title and abstract screening, and then full-text screening by 2 independent reviewers. Data regarding the studies, intervention characteristics, their theoretical basis (eg, use of behavior change frameworks such as the COM-B [Capability, Opportunity, Motivation-Behavior] model or the social cognitive theory), evaluation methods, and outcomes will be extracted into a predetermined form. A meta-analysis will be conducted on eligible studies (reporting control group comparisons) to synthesize evidence of their effectiveness, and the remaining quantitative data will be descriptively analyzed.

**Results:**

The review is expected to start in December 2025 and to be submitted for publication by the end of 2026.

**Conclusions:**

This review will synthesize evidence on the theoretical basis underpinning mHealth interventions for enhancing physical activity, healthy eating, and weight loss and generate new insights into how particular behavior change techniques can best support intended outcomes. This will help guide the development of more impactful mobile-based interventions to support healthy behaviors that are better able to reduce the risk factors for chronic health conditions.

**Trial Registration:**

PROSPERO CRD42024602819; https://www.crd.york.ac.uk/PROSPERO/view/CRD42024602819

**International Registered Report Identifier (IRRID):**

PRR1-10.2196/72664

## Introduction

### Background

The global rise of noncommunicable diseases is increasing pressure on health care systems and economies, alongside the impact on individuals’ quality and length of life [1,2]. Obesity is a risk factor in many noncommunicable diseases, and weight management through healthy behaviors, including healthy eating and physical activity [3,4], is key to prevention [5-7]. Digital health interventions (DHIs) offer opportunities to support greater numbers of at-risk individuals than traditional, offline weight management interventions [8], but evidence of DHI effectiveness, particularly over sufficient durations to be clinically meaningful, is limited [9,10]. Key barriers to digital health impact include lack of engagement [11-16] and lack of digital access and/or literacy, which can also widen health inequities [8]. Increasing evidence suggests that theory-based mobile-based interventions for health behavior changes could enhance their effectiveness in various aspects such as user engagement [17], self-efficacy [18], patient self-management [19], and attitudes toward physical activity [20]. The influence of specific theories and behavior change techniques (BCTs) on the effectiveness of mobile-based interventions is still unclear. This review will provide an update to previous work [21] exploring the theoretical basis and BCTs (or “active ingredients”[22]) included in the interventions, as well as their effectiveness through a meta-analysis. Given their important roles in chronic disease prevention, we will focus on physical activity, healthy eating, and weight loss [23,24].

Incorporating behavioral theory into the development of DHIs can improve their impact on health behaviors [25], but many DHIs are developed without a theoretical basis [26,27]. Potential reasons for this lack include theory complexity, with some designers citing that they are inaccessible to nonexperts of behavior change theory [26] and the diversity of behavioral theories available. For example, Michie et al’s (2011) [28] COM-B (Capability, Opportunity, Motivation-Behavior) model, which posits that behavior change requires a combination of considerations of individuals’ capability, opportunity, and motivation, is associated with the taxonomy of BCTs [22]. BCTs are increasingly being deliberately incorporated in digital behavior change interventions [29-31], but the diversity of behavioral theories can lead to confusion about which BCTs may be most useful to include in different health contexts [32]. For example, the social cognitive theory, commonly used as a theoretical basis in DHIs [21], states that goal setting is key to behavior change [33], which would suggest prioritizing the goal-setting BCT within interventions [34]. However, there is substantial evidence that people’s intentions do not necessarily align with their actual behavior, particularly in interventions targeting long-term healthy lifestyle behaviors such as physical activity and healthy eating [35-38].

Behavioral theories such as the dual process theory [39] have been applied to attempt to explain this gap, with researchers positing that conscious and automatic processes (ie, habits) must be activated for sustained behavior change. Evidence suggests that web-based physical activity interventions targeting habit formation effectively support sustained behavior change [40], supporting this theory in the context of mobile health (mHealth) intervention development. Other theories that emphasize the role of cognition (eg, the cognitive continuum theory, a theory of decision-making suggesting that decision-making involves both intuitive, ie, automatic and analytical thinking, influenced by task complexity and familiarity [41] and the cognitive load theory, which posits that the learning is impacted by the mental effort required) are also potentially applicable to the behavior change intervention design [42]. Interventions aiming to minimize the cognitive load on users may be more successful at facilitating behavior change by reducing barriers to engagement. Digital-specific models such as the Technology Acceptance Model and the Unified Theory of Acceptance and Use of Technology emphasize the importance of the solution’s ease of use and perceived usefulness in facilitating engagement [43,44]. While hardly exhaustive, and drawn predominantly from the cognitive rather than wider social domains of behavioral sciences, these examples highlight the wide variety of theories that could be used to support DHI design for sustainable behavior change.

The effectiveness of mobile app–based interventions in promoting health behaviors has been explored in systematic reviews and meta-analyses, with mixed findings. For example, significant effectiveness was not found in mobile app–based interventions for physical activity in healthy adults aged 18 to 35 years [45]. In contrast, recent meta-analysis studies revealed that mobile apps can lead to significant improvements in behavioral and health outcomes in various population groups, such as increasing total physical activity and reducing BMI in children and adolescents [46], as well as various weight-related outcomes in adults with overweight and obesity (reduced weight, BMI, waist circumference, fat mass, and diastolic blood pressure) [47]. An umbrella review on mHealth interventions examined the impact of mHealth across various health outcomes, some of which involved weight loss and physical activity [48]. There is a lack of exploration of the behavioral frameworks underlying the interventions, and although the use of specific BCTs in mHealth interventions was examined in one review [49], it focused on frequency and effectiveness in interventions that did not use mobile technologies. This highlights the need for further research to explore the association between these outcomes and the use of specific BCTs. Knowing the effects of BCTs on behavior change outcomes could provide valuable insights to guide the development of more effective mHealth apps. The impact of mobile-based interventions on healthy eating was also not explored in previous reviews, leaving a gap in the literature. A healthy diet has a significant positive impact on weight loss [50]. Exploring these aspects together may yield a better understanding of the effectiveness of mobile app interventions in promoting health behavior change.

We previously conducted a systematic review in 2019 (literature searched between 2014-2019), aiming to evaluate the effectiveness of mHealth apps in improving physical activity, diet, drug and alcohol use, and mental health and to explore the use of BCTs in these apps and found limited evidence supporting their effectiveness [21]. However, while the broad scope provided a comprehensive overview, its variety of outcomes meant we could not conduct a meta-analysis, which was a key limitation of the review. In addition, app development accelerated exponentially during and after COVID-19, which occurred after our review was conducted [51,52]. This highlights a need to update the previous review with a more specific focus to enable a meta-analysis and the inclusion of more recent literature to synthesize the developments in the field.

### Objectives

The primary aim of this review is to update a previous systematic review that evaluated the effectiveness of mobile apps for health behavior change [21], with the addition of a meta-analysis to quantitatively synthesize effectiveness. To ensure a more focused approach, we will narrow the scope only to include studies on weight management interventions. Secondary objectives include exploring behavioral theories and BCTs incorporated in the apps and synthesizing the apps’ feasibility, usability, and user experience. Three main research questions will guide this review. First, what is the effectiveness of mHealth apps at improving and maintaining health behavior change with respect to healthy eating and physical activity? Second, what behavioral theories and BCTs are being incorporated in mHealth app design to support engagement with these behaviors and which are the most effective at promoting behavior change? Third, what is the feasibility, usability, and user experience associated with these apps? By providing a comprehensive synthesis of these aspects, we aim to inform the development of mHealth apps for weight management, particularly concerning behavior change theories, models, and frameworks.

## Methods

### Study Design

This protocol has been prepared in line with the PRISMA-P (Preferred Reporting Items for Systematic Reviews and Meta-Analysis Protocols) guidelines (see Multimedia Appendix 1) and the search strategy structured with reference to the PICOS (Population, Intervention, Comparator, Outcome, and Study) framework [53]. This review was registered on PROSPERO (CRD42024602819). A systematic review design was chosen because the primary objective of the review will be to synthesize the effectiveness of mHealth apps. Although the secondary objectives are more exploratory, they will primarily be used to contextualize the evidence and synthesized using systematic methods [54].

### Search Strategy

We will search 9 databases: PubMed, Embase, CINAHL, APA PsycINFO, Cochrane Library, SPORTDiscus, SCOPUS, Web of Science, and Science Direct. These have been chosen, as they comprehensively cover the fields of medicine, sport/exercise, nutrition, and computing/artificial intelligence.

The eligibility criteria and search terms are shown in Table 1. A literature review identified keywords and MeSH (Medical Subject Headings) terms relating to evaluations of mHealth apps targeting weight loss, healthy eating, and physical activity outcomes. Keywords and MeSH terms were expanded through database searching and grouped into themes. The 3 themes resulted in the following search structure: mHealth (MeSH OR keywords) AND weight-related behaviors (MeSH OR keywords) AND evaluation (keywords).

The full search string used for an initial search of PubMed and the number of references returned are included in Multimedia Appendix 2.

**Table 1:** Eligibility criteria, search themes, MeSHa terms, and keywords structured according to the PICOS (Population, Intervention, Comparator, Outcome, and Study) framework.

Element	Eligibility criteria	MeSH terms	Keywords
Population	The population will include people of any age (children, adolescents, or adults).	N/A^b^	N/A
Intervention	mHealth^c^ interventions targeting physical activity, sedentary and healthy eating behaviors, and weight loss will be included.	Cell Phone OR Telemedicine OR Mobile Applications	Smartphone OR “mobile phone” OR “mHealth” OR “mobile health” OR App OR apps OR “mobile app*” OR “smartphone app*” OR “digital health”
Comparator	Not required for inclusion in the systematic review Studies with control group comparisons will form a subset for meta-analysis	N/A	N/A
Outcomes	Outcomes include weight loss, healthy eating, physical activity, health behavior change, and engagement.	Health Behavior OR Exercise OR Weight Loss OR Obesity (diet therapy, prevention & control, rehabilitation, therapy) OR Nutrition Therapy OR Diet OR Sedentary Behavior	overweight OR obesity OR obese OR “weight loss” OR “weight management” OR “weight reduction” OR “weight maintenance” OR “maintaining weight” OR “ideal weight” OR “body fat” OR “waist circumference” OR “body mass index” OR BMI OR “healthy eating” OR diet OR nutrition OR calori* OR “meal plan” OR “eating behav*” OR cook* OR food* OR fruit* OR veg OR vegetable* OR “physical activit*” OR “moderate to vigorous” OR “active living” OR exercise OR fitness OR “active transport” OR sedentary OR physical inactivity OR walk* OR “behavior change” OR “behavior change” OR Exercise ADJ3 (increase or start or maintain*)) OR “physical activity” OR “Weight loss” OR “healthy weight” OR “five a day” OR “diet” OR “nutrition” OR ((Maintenance OR maintain* OR achiev* Or retain*) ADJ4 (weight goal OR “weight loss” OR “goal weight” OR BMI))
Study types	Studies in English, published since 2019 evaluating the impact of mHealth interventions supporting intention or habit on the specified outcomes will be included. Protocols, conference abstracts, reviews, and other studies that do not contain an evaluative component will be excluded.	N/A	effective* OR efficacy OR impact* OR evaluat* OR study OR test OR trial OR Feasibility OR usability OR “outcome*” OR acceptability OR adherence OR “effectiv*” OR “adoption” OR “assess*”

^a^MeSH: Medical Subject Headings.

^b^N/A: not applicable.

^c^mHealth: mobile health.

### Inclusion Criteria

All types of studies evaluating the impact of an mHealth intervention on weight-related, healthy eating, or physical activity outcomes will be eligible for inclusion. Given that digital health is a rapidly advancing field, particularly after COVID-19 [55,56], and this review is related to the current state of mHealth interventions, it will include studies published since 2019. This time frame allows for a comprehensive update to Milne-Ives et al’s [21] systematic review, which included papers from 2014 to 2019. The subset of studies reporting control group comparisons will additionally be eligible for inclusion in the meta-analysis.

### Exclusion Criteria

References that describe the development or implementation of interventions without reporting the impact on weight loss, healthy eating, or physical activity outcomes will be excluded, as this study focuses on the interventions’ impact on outcomes. Studies of apps that were designed for use by health care professionals or those that focus on mobile phones or other digital technology but not apps (eg, email, SMS text messaging interventions) will also be excluded. Studies not reported in English will be excluded due to the language capabilities of the research team. Where full text is unavailable, references will be excluded. Reviews, conference abstracts, surveys, and case studies will also be excluded, as our focus is on original, evaluative research concerning digital behavior change interventions.

### Screening and Study Selection

References will be compiled and duplicates removed in EndNote 21 software. A series of keyword search passes will be conducted to exclude ineligible references. The remaining references will be uploaded to Rayyan to facilitate manual screening of titles and abstracts by 2 independent reviewers, after which full-text screening by 2 independent reviewers will be completed to arrive at a final set of included references. Disagreements between reviewers will be resolved by discussion and consultation with a third reviewer if required. EndNote-automated screening passes will be recorded for reference, and the outcomes of each stage of the process will be recorded in a PRISMA flow diagram; all details will be available in the review report.

### Data Extraction

Table 2 shows the study data to be extracted for analysis. As with the screening, 2 reviewers will extract the data independently, with disagreements discussed until consensus and a third reviewer involved if necessary.

**Table 2:** Study data to be extracted.

Information area	Data to be extracted
Study characteristics	Publication year Country of study Study type Study duration, including intervention duration and follow-up timepoint or timepoints Population demographics Sample size Comparator
Intervention characteristics	Target health behavior or behaviors Intervention focus (ie, healthy eating, physical activity, or both) Whether BCTsa explicitly described in paper (yes/no) BCTs employed (list - coded based on behavior change ontology [57]) Behavioral theory or theories’ basis Name of the app Developers Platform Design features (eg, feedback provision, notifications, monitoring)
Evaluation	What outcomes were reported Impact on health outcomes (eg, change in BMI, body weight, body fat, waist circumference, blood pressure, cholesterol levels)b Impact on behavioral outcomes (eg, change in calorie intake, intake of fruit/vegetables, intake of processed foods, step count, metabolic equivalent of tasks, minutes of exercise per week, minutes of physical activity by level, sedentary periods)b Engagement outcomes (eg, engagement self-report scales, app usage data) Feasibility Usability User experience/satisfaction Other outcomes Stated strengths and limitations

^a^BCT: behavior change technique.

^b^Quantitative outcomes potentially eligible for meta-analysis if sufficient data are available.

### Quality and Risk of Bias Assessments

Quality and risk of bias will be assessed by 2 reviewers, any disagreements discussed, and a third reviewer consulted if a consensus cannot be reached. Randomized controlled trials will be assessed using the revised Cochrane Risk of Bias tool [58]. Nonrandomized studies will be assessed using the Newcastle-Ottawa Scale [59].

### Data Analysis and Synthesis

Based on initial literature searches, it is anticipated that a subset of the studies will be suitable for inclusion in a meta-analysis and that these studies will be heterogeneous in their outcome measures. A random-effects meta-analysis model for overall effect size, using Hedge *g* to calculate the standardized mean difference (with a 95% CI), and τ^2^ to measure heterogeneity will be employed. SPSS software (version 29; IBM Corp) will be used for the statistical analysis. The meta-analysis will aim to synthesize the effectiveness of the included apps, and separate analyses will be conducted for the different outcome measures identified (eg, change in BMI or body weight, change in waist circumference, change in calorie intake). Missing data will be handled on a case-by-case basis, following the Cochrane Handbook for Systematic Reviews of Interventions [60]. Where possible, original investigators will be contacted to request missing data (particularly for missing studies, to mitigate publication bias). Based on the data found, assumptions as to whether they are missing at random will be made and clearly outlined, and risk of bias assessments will take this into account. Additionally, sensitivity analyses will be performed based on the data found to assess the impact of reasonable changes in the assumptions made. A descriptive analysis will be conducted to summarize the quantitative results of studies not suitable for inclusion in the meta-analysis. Data about the use of BCTs and the feasibility, usability, and user experience of the apps will be summarized following Popay et al’s (2006) [61] narrative synthesis guidance, chosen as it would enable us to integrate a variety of qualitative and quantitative research findings. The strengths and limitations of the identified studies and areas for future research and development will be discussed.

## Results

Work on this review has not yet commenced as of October 3, 2025. Data collection will be completed by December 2025, and findings will be submitted for publication by the end of 2026. Data used for analysis in this review will be deposited in the Open Science Framework [59] upon completion of the study for transparency.

## Discussion

### Summary of the Anticipated Findings

This review will provide a synthesis of the effectiveness of mobile apps for health behavior change in terms of healthy eating and physical activity. We will also examine the apps’ feasibility, usability, and user experience, and explore the behavioral theories and BCTs used in developing them. By synthesizing all this information in one place, we hope to explore how the use of theory in DHI development might best contribute to their effectiveness, particularly in the long term. The findings of this review will update existing syntheses of similar apps, providing a focused summary of apps for physical activity and diet change. Although the mixed findings in previous reviews preclude a hypothesis, we anticipate that apps incorporating specific BCTs may show increased evidence of effectiveness.

### Dissemination and Stakeholder Engagement

The review will be disseminated via publication in a peer-reviewed journal and via an online newsletter distributed by the research group. We will also distribute a summary of the research through Newcastle University’s networks, including the Behavioral Sciences and Psychology Theme.

### Comparison to Previous Work and Future Directions

By synthesizing the effectiveness of DHIs for behavior change through a meta-analysis and contextualizing this via an exploration of user experience, usability, feasibility, and the use of BCT, we aim to enable the development of apps grounded in theory that enable long-term engagement and sustained change. Although previous research has synthesized the effectiveness of similar apps [46,47], none have conducted a comprehensive assessment of the qualitative and quantitative outcomes as well as the theoretical grounding, and few have focused on diet in addition to physical activity. Our previous review attempted to analyze this comprehensively, its broad scope meant a meta-analysis was infeasible, and an update is needed as this was conducted in 2019. We expect that this review will provide better insights into such apps, particularly adding to the knowledge of their impact on healthy diet, and their use of BCTs to enable these changes. This review will highlight for health care professionals and app developers the characteristics of mHealth interventions that might lead to sustained change, inform future development, and allow individuals to choose effective DHIs to use and recommend, if any are identified.

### Limitations

Due to the language capability of the research team, this study will not include studies in languages other than English, which may miss relevant studies in other languages. Studies published before 2019 will also be excluded to avoid duplicated efforts in previous studies and to provide a more recent update on the evidence. Conference abstracts and grey literature will be excluded to focus the review on studies that are likely to be higher quality, but there is a risk that relevant information may be missed.

### Conclusions

This review will contribute to the body of knowledge around the theoretical foundations for mHealth interventions, particularly those related to physical activity, healthy eating, and weight loss, by synthesizing recent mHealth evaluations. To address the gap in previous reviews, we will explore the associations between these BCTs and the effectiveness of mHealth interventions through a meta-analysis. This will offer new insights about which technique is more effective for specific outcomes. Such understanding can contribute to the design of more impactful mHealth interventions in the future.

## Appendix

Multimedia Appendix 1 PRISMA-P checklist.

Multimedia Appendix 2 Sample search on PubMed.

## Abbreviations

BCT: behavior change technique
COM-B: Capability, Opportunity, Motivation-Behavior
DHI: digital health intervention
MeSH: Medical Subject Headings
mHealth: mobile health
PICOS: Population, Intervention, Comparator, Outcome, and Study
PRISMA-P: Preferred Reporting Items for Systematic Reviews and Meta-Analysis Protocols
