# Supplementary material for: Effectiveness and Theoretical Foundations of mHealth Apps for Physical Activity, Healthy Eating, and Weight Loss: Protocol for a Systematic Review and Meta-Analysis
Source: JMIR Res Protoc. 2026 Feb 17;15:e72664. doi: 10.2196/72664 (PMC12957943; doi:10.2196/72664)
Supplement: Multimedia Appendix 2 [file resprot_v15i1e72664_app2.docx]

**Multimedia Appendix 2: Sample search on PubMed**

| **Database (date of search)** | **Search string** | **Number of references** |
| --- | --- | --- |
| Pubmed (17 Oct) | ("digital health"[MeSH Terms] OR "telemedicine"[MeSH Terms] OR "mHealth"[Title/Abstract] OR "ehealth"[Title/Abstract] OR "telehealth"[Title/Abstract] OR "mobile app*"[Title/Abstract] OR "smartphone app*"[Title/Abstract] OR "phone app*"[Title/Abstract] OR "cellphone app*"[Title/Abstract]) AND ("overweight"[MeSH Terms] OR "obesity"[MeSH Terms] OR "weight loss"[MeSH Terms] OR "diet, healthy"[MeSH Terms] OR "exercise"[MeSH Terms] OR "sedentary behavior"[MeSH Terms] OR "caloric restriction"[MeSH Terms] OR "weight management"[Title/Abstract] OR "body fat"[Title/Abstract] OR "waist circumference"[Title/Abstract] OR "healthy eating"[Title/Abstract] OR "diet"[Title/Abstract] OR "nutrition"[Title/Abstract] OR "physical activity"[Title/Abstract] OR "MVPA"[Title/Abstract] OR "fitness"[Title/Abstract] OR "sedentary"[Title/Abstract]) AND ("evaluat*"[Title/Abstract] OR "effective*"[Title/Abstract] OR "impact*"[Title/Abstract] OR "efficacy"[Title/Abstract] OR "intent*"[Title/Abstract] OR "habit*"[Title/Abstract]) | 5265 |
